# Establishment and Characterization of a New Human Intrahepatic Cholangiocarcinoma Cell Line LIV27

**DOI:** 10.3390/cancers14205080

**Published:** 2022-10-17

**Authors:** Xiwei Ding, Kais Zakharia, Catherine D. Moser, Nellie A. Campbell, Chunling Hu, Nataliya Razumilava, Roongruedee Chaiteerakij, Hassan M. Shaleh, Patricia T. Greipp, Rondell P. Graham, Xiaoping Zou, Vishal S. Chandan, Lewis R. Roberts

**Affiliations:** 1 Department of Gastroenterology, Nanjing Drum Tower Hospital, The Affiliated Hospital of Nanjing University Medical School, Nanjing 210000, China; 2 Division of Gastroenterology and Hepatology, University of Iowa, Iowa City, IA 52242, USA; 3 Aurora St. Luke’s Medical Center, GI Associates, Milwaukee, WI 53215, USA; 4 Mayo Clinic and Mayo Clinic Cancer Center, Division of Gastroenterology and Hepatology, College of Medicine and Science, Rochester, MN 55905, USA; 5 Division of Gastroenterology, Department of Medicine, Faculty of Medicine, Chulalongkorn University and King Chulalongkorn Memorial Hospital, Bangkok 10330, Thailand; 6 Department of Laboratory Medicine and Pathology, Mayo Clinic, Rochester, MN 55905, USA; 7 Department of Pathology and Laboratory Medicine, School of Medicine, University of California, Irvine, CA 92697, USA

**Keywords:** cholangiocarcinoma, LIV27, cell line, primary sclerosing cholangitis, short tandem repeat

## Abstract

**Simple Summary:**

Cholangiocarcinoma (CCA) is usually diagnosed at a late stage and has a poor prognosis. Cell lines serve as useful models for testing scientific questions in vitro and in vivo. To aid scientific discovery for the purpose of improved early detection and treatment, we developed a CCA cell line, designated LIV27, from a surgically resected intrahepatic CCA in a Caucasian patient with primary sclerosing cholangitis (PSC). LIV27 has high tumorigenicity in nude mice and expands the availability of intrahepatic CCA cell lines.

**Abstract:**

Cholangiocarcinoma (CCA) is a highly lethal cancer arising from the biliary tract epithelium. The cancer biology of this neoplasm is not well understood. To date, only a few CCA cell lines have been reported, which were mostly developed from Asian patients. In this study, we report and characterize a new intrahepatic CCA cell line, LIV27, derived from a surgically resected tumor in a 67-year-old Caucasian woman with primary sclerosing cholangitis (PSC). LIV27 cells grow well in collagen-coated flasks or plates with a doubling time of 57.8 h at passage 14. LIV27 cells have high tumorigenicity in nude mice and stain positive for CK7 and CK19, markers that differentiate CCA from hepatocellular carcinoma. Karyotype analysis showed that LIV27 is aneuploid. We established a single-locus short tandem repeat profile for the LIV27 cell line. This newly established cell line will be a useful model for studying the molecular pathogenesis of, and developing novel therapies for, cholangiocarcinoma.

## 1. Introduction

Cholangiocarcinoma (CCA) is an aggressive malignancy arising from the biliary tract. This tumor is highly lethal, as the 5-year survival rate has not increased substantially over the past 3 decades, despite advances in diagnostic imaging and therapeutic approaches [1]. CCA is currently classified based on anatomic location into three distinct subsets: intrahepatic (iCCA), perihilar (pCCA), and distal CCA (dCCA). These subsets are different in terms of their genetics, clinical presentation, management, and outcomes. In the highest-incidence countries in Asia, particularly Thailand, liver fluke infestation of the bile ducts is a major predisposing factor for CCA [2]. In populations of European descent, primary sclerosing cholangitis (PSC) is associated with a high risk of developing CCA [3]. Other major risk factors include congenital biliary tract malformations and biliary stone disease. Toxins, including dichloromethane (used as a cleaning solvent in the offset printing industry), have also been associated with an increased risk of CCA [4]. Notably, the incidence of intrahepatic CCA is increasing in many industrial countries, including the USA, Germany, and Australia [5,6,7,8]. The molecular pathogenesis and biology of CCA are poorly understood [9]. Genomic studies have revealed the substantial prevalence of oncogenic alterations of the fibroblast growth factor (FGF) pathway, notably FGF receptor 2 (FGFR2) fusions, and mutations of the isocitrate dehydrogenase (IDH) 1 and 2 genes in iCCA [10,11]. The treatment for CCA has evolved from a situation in which surgical resection and conventional cytotoxic chemotherapies were the only treatment options to one that has witnessed the recent approval of precision-targeted therapies, such as FGF receptor inhibitors [12,13,14,15] and IDH inhibitors [16,17,18]. Since PSC is one of the most important risk factors for CCA, we had a strong interest in developing CCA cell lines in patients with PSC [19,20,21].

Cancer cell lines are very useful models for studying tumor biology and therapy. Progress in our understanding of the molecular mechanisms of CCA has been limited by the relative lack of tumor cell lines. Although several CCA cell lines have been reported in the literature to date, most were established in Asian patients and most were from extrahepatic CCA [22,23,24,25,26,27,28,29,30,31,32,33]. In addition, to the best of our knowledge, no PSC-associated CCA cell lines have been reported. In this study, we established and characterized a new human intrahepatic CCA cell line, LIV27, which was derived from a Caucasian female patient with PSC. This newly established cell line provides a useful model for the study of intrahepatic CCA, especially PSC-associated CCA.

## 2. Materials and Methods

### 2.1. Patient Information

A 67-year-old Caucasian woman presented with a 4-month history of nausea and weight loss. She was found to have a 5.4 × 5.3 × 5.2 cm tumor in the left hepatic lobe upon imaging. She did not have jaundice, and the total bilirubin level was normal. The carbohydrate antigen 19-9 (CA19-9) was 136 U/mL (normal ≤ 55 U/mL) at the time of diagnosis. Hepatitis B virus (HBV) and hepatitis C (HCV) virus serologies were negative. She had a history of PSC with stage 3 (of 4) liver fibrosis, ulcerative colitis, Caroli’s disease, hypothyroidism, and rheumatoid arthritis. Left lobe partial hepatectomy was performed. No portal vein or distant metastasis was detected at the time of surgical resection. A histologic exam of the resected tumor showed mass-forming intrahepatic cholangiocarcinoma. There was no evidence of vascular invasion. The uninvolved liver parenchyma showed concentric periductal fibrosis (classic “onion skin”) consistent with PSC. Metastatic carcinoma was identified in one of two hilar lymph nodes. The TNM tumor stage was IIIB (pT1bN1cM0) according to the latest AJCC staging system. The primary tumor was used for the isolation of cancer cells. The study was approved by the Mayo Clinic Institutional Review Board.

### 2.2. NOD/SCID/Il2rg Null (NSG) Mouse Implantation and Cell Line Establishment

Fresh cholangiocarcinoma tumor tissue obtained directly during surgery was placed in Minimum Essential Media (MEM) medium (GIBCO) and delivered to the laboratory. Under aseptic conditions, the tumor tissue was washed with phosphate-buffered saline (PBS). The tissue was minced into very fine pieces in a sterile dish with a small amount of PBS. A total of 0.2 mL minced tumor tissue was mixed with 0.1 mL of Matrigel (BD Biosciences) and injected into the flank of an NSG mouse to establish a patient-derived xenograft (PDX). Three NSG mice were injected, and all three developed subcutaneous tumors within 10 weeks. The tissues from these mice were labeled as 1st generation NSG tumors. The tumors were removed and divided into several pieces for different methods of processing. One piece was used for serial passage into three more NSG mice, following the procedure described above. One piece was formalin-fixed and paraffin-embedded. An H&E slide made from this paraffin-embedded tissue was reviewed by a hepatobiliary pathologist at the Mayo Clinic (V.S.C).

Tissue from 2nd- and 3rd- generation NSG mouse PDXs was used to develop explant cell cultures. The tissue was placed in a 100 mm dish and minced very finely in PBS. The tissue was then filtered in PBS through a 70 μm cell strainer. The cells were spun down, resuspended in 5% platelet lysate and DMEM-F12 media supplemented with 0.393 μg/mL dexamethasone, 0.1 μg/mL insulin, and 1× anti-mycotic/anti-biotic (Invitrogen, Waltham, MA, USA), and seeded into a collagen-coated flask. The cells were maintained at 37 °C in the presence of 5% CO_2_. The medium was changed twice a week. The cells were sub-cultured when they reached 70–80% confluence.

### 2.3. Morphologic Examination and Growth Kinetics

The cultured cells were routinely monitored and photographed by phase-contrast microscopy. Cells of passage 14 were studied to estimate the population doubling time. On day 0, 1 × 10^5^ cells were plated in duplicates onto 6-well plates. The cell culture medium was changed every two days. Cells were detached from the wells with trypsin-EDTA, and the average number of viable cells from two wells was counted every 24 h in a hemacytometer chamber after staining with trypan-blue dye. The cells were counted for up to 9 days. The growth curve was plotted, and the doubling time of the cell population was estimated during the logarithmic growth phase. We also tested the cell viability of the LIV27 cells at passages 12 and 21. Briefly, 2 × 10^4^ LIV27 cells were seeded per well on 96-cell plates. The XTT Cell Viability Kit (Cell Signaling Technology, Danvers, MA, USA) was used to test the cell viability.

### 2.4. Tumorigenicity in Nude Mice

Animal protocols were reviewed and approved by the Institutional Animal Care and Use Committee at the Mayo Clinic. Then, 6–8-week-old female nude mice were purchased from the National Cancer Institute at Frederick, MD. The cells of passage 8 were prepared to determine their tumorigenicity in the nude mice. Then, 1 × 10^6^ LIV27 cells were suspended in 0.1 mL PBS, mixed with 0.1 mL Matrigel, and injected subcutaneously into the right flanks of 10 nude mice. The tumor volume and body weight were recorded every 3–5 days. The tumor volume was calculated using the equation: Volume = L × S^2^/2, where L and S represent the longest and shortest diameter of the tumor, respectively. Tumor tissue was excised, fixed in 10% formalin, and processed for routine histopathological examinations.

### 2.5. Immunofluorescence

The cells were fixed with methanol/acetone for 20 min, washed with PBS, incubated with 0.1% Triton-X-100 for 2 min, washed again, and then blocked for 1 h in 5% goat serum. The cells were then incubated with antibodies against CK7 (sc-53263, 1:50, Santa Cruz Biotechnology, Dallas, TX, USA), CK19 (LS-B3148, 1:50, LifeSpan Biosciences, Seattle, WA, USA), EPCAM (MA5-12442, 1:50, Thermo Fisher Scientific, Waltham, MA, USA), or the IgG control overnight at 4 °C. The cells were washed and incubated with secondary antibodies (1:200) for 1 h at room temperature and then washed again and coverslipped with Prolong Gold Antifade Mountant combined with DAPI. The cells were examined by confocal microscopy (LSM 780, Carl Zeiss, Oberkochen, Germany).

### 2.6. Western Blot

Equivalent amounts of protein were separated on a 4–15% Tris-HCl gel and transferred to PVDF membranes. The membranes were probed with the appropriate primary antibodies. The blots were incubated with horseradish-peroxidase-conjugated secondary antibodies. Then, signals were visualized using the HyGLO HRP detection kit. GAPDH was used as the loading control.

### 2.7. Karyotyping and STR Profiling

The LIV27 cells were karyotyped by the Mayo Clinic Core Facility. Cells were plated at approximately 1–2 × 10^6^/75 cm^2^ flask. After 48 h, the cells were exposed to 0.1 μg of colchicine (Sigma, St. Louis, MO, USA) at 37 °C for 3 h. The cells were then harvested by trypsinization, incubated for 20 min at room temperature with a hypotonic solution (75 mM KCL), and fixed with methanol: acetic acid (3:1). Slides were prepared and stained with Giemsa. G banding was performed in order to analyze the chromosomal aberrations. Representative chromosome sets were photographed and karyotyped. Short tandem repeat profiling (STR) was performed on the LIV27 cells by GENEWIZ (South Plainfield, NJ, USA) in September 2014.

### 2.8. Mycoplasma Detection

The MycoAlert™ Mycoplasma Detection Kit (Lonza) was routinely used to detect any mycoplasma contamination following the manufacturer’s instructions.

## 3. Results

### 3.1. Establishment of the LIV27 Cell Line

**Figure 1:**
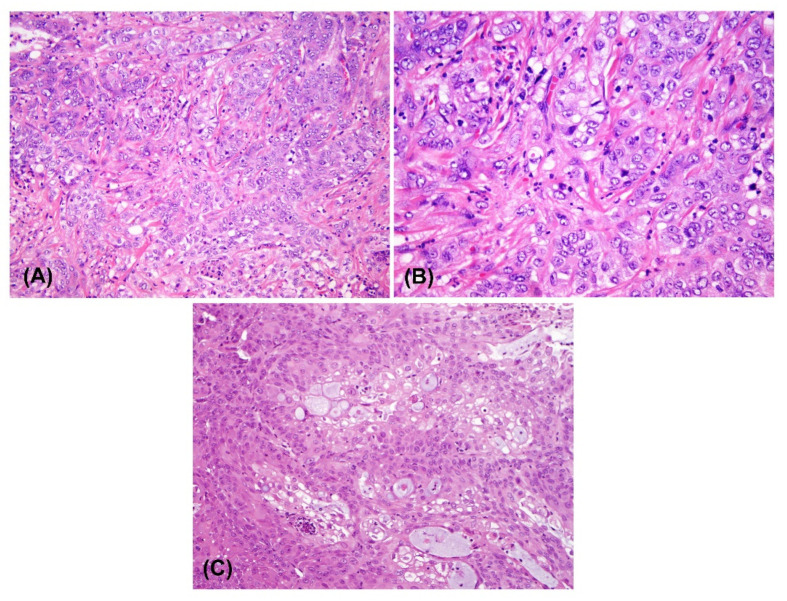
H&E staining of the original tumor and tumor from a patient-derived xenograft (**A**) and (**B**) histopathological analysis of the specimen indicates moderate differentiated cholangiocarcinoma. (**A**) The 200× magnification and (**B**) 400× magnification. (**C**) Histopathological analysis of the LIV27 patient-derived xenograft shows a similar histology to the original tumor but with less fibrous stroma (200× magnification).

The histologic exam of the resected primary tumor showed moderate differentiated cholangiocarcinoma (Figure 1A,B). Fresh cholangiocarcinoma tumor tissue obtained directly during surgery was used to establish a PDX. Three NSG mice were injected, and all three developed subcutaneous tumors within 10 weeks. The histopathological analysis of the PDX specimen showed similar histology with the original tumor, except for the fact that there were less fibrous stroma (Figure 1C). Tissue from the 2nd- and 3rd-generation NSG mouse PDXs was used to develop explant cell cultures. Contaminating fibroblasts were periodically removed with a cell scraper and by differential trypsinization. Finally, the malignant cells were sorted by flow cytometry using the epithelial cell marker EPCAM in order to further remove the fibroblasts.

### 3.2. Morphological Analysis and Population Doubling Time

**Figure 2:**
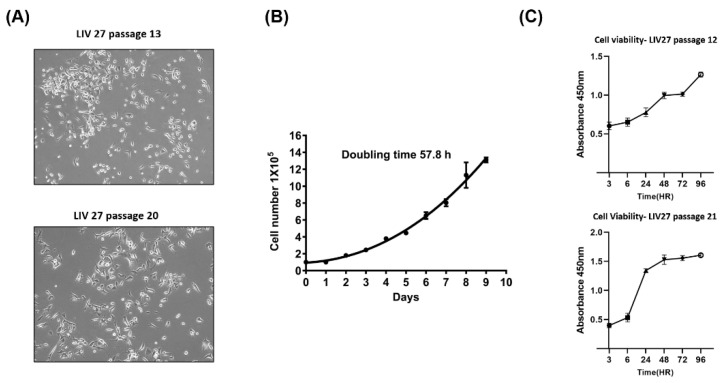
Growth curve of LIV27 cells in vitro. (**A**) Cell morphology was observed by phase-contrast microscopy at passages 13 and 20. The cell line exhibited spindle- to polygonal-shaped morphology. (**B**) Cumulative growth curve of LIV27 cells. Data shown are mean ± SE. (**C**) The cell viability curves of LIV27 cells at passages 12 and 21. Data shown are mean ± SE.

On phase-contrast microscopy, the cultured LIV27 cells grew as an adherent monolayer with characteristic epithelial morphologic features. The cells were spindle- or polygonal-shaped and of various sizes (Figure 2A). After thawing, the cryopreserved cells could be propagated in culture without noticeable changes in their growth and morphology. The population doubling time of the LIV27 cell line was approximately 57.8 h at passage 14 (Figure 2B). The cell viability curves were similar at passages 12 and 21 (Figure 2C).

### 3.3. Tumorigenicity in Nude Mice

**Figure 3:**
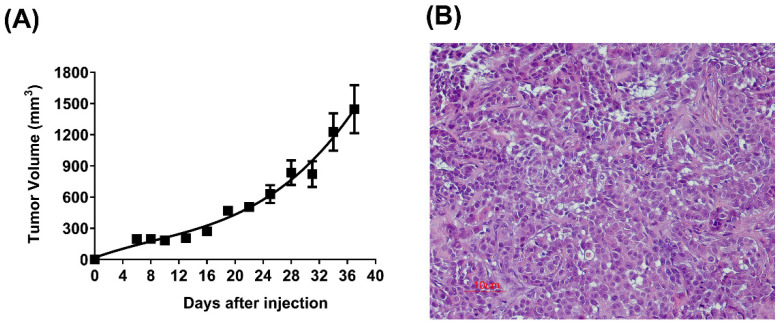
Growth curve of LIV27 cells in vivo. (**A**) Tumorigenicity test of LIV27 cells in nude mice showing a mean tumor volume reaching 1400 mm^3^ at 37 days following the subcutaneous injection of 1 × 10^6^ LIV27 cells. Data shown are mean ± SE (*n* = 10). (**B**) Histology of the subcutaneous tumor showing the compact growth of tumor cells with little fibrous stroma (H&E, 200× magnification).

The LIV27 cells were highly tumorigenic in the athymic nude mice. We observed that 4–6 days after the sub-cutaneous injection of 1 × 10^6^ cells per mouse, visible tumors developed in all 10 nude mice at the site of inoculation. The tumor nodules reached a mean dimension of 1.4 cm in about 5 weeks (Figure 3A). The histological examination of the xenotransplanted tumor showed a similar histology to the original tumor, except for the fact that there were less fibrous stroma (Figure 3B).

### 3.4. Expression of Biliary Marker

**Figure 4:**
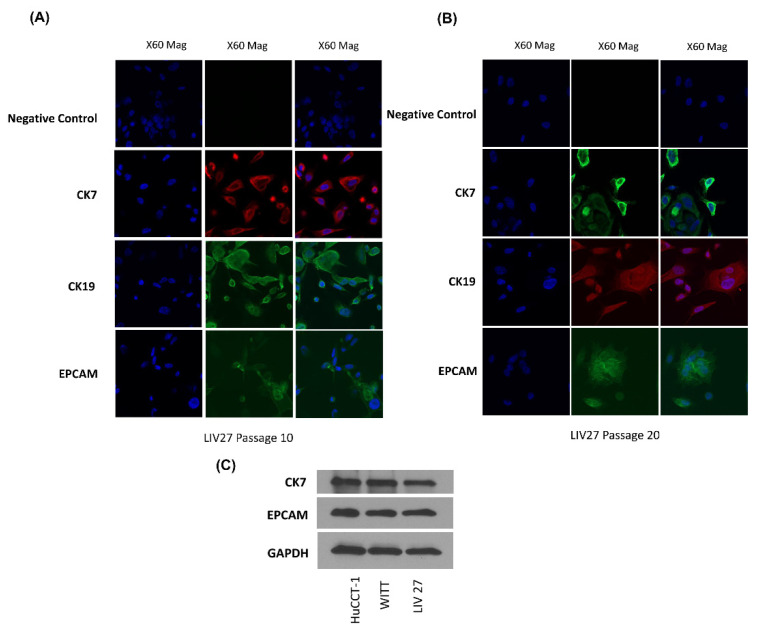
(**A**,**B**) Expression of the epithelial markers CK7, CK19, and EPCAM versus the negative control antibody in LIV27 cells detected by immunofluorescence. The images were captured at 60× magnification. (**C**) Expression of the biliary epithelial marker CK7 and epithelial marker EPCAM in three different cholangiocarcinoma cells detected by Western blot. All the whole western blot figures can be found in the Supplementary Materials.

The expression of the epithelial markers CK7, CK19, and EPCAM was studied in the established cultures by immunofluorescence. LIV27 showed positive staining for human CK7, CK19, and EPCAM, distinguishing the bile duct epithelial cells from hepatocytes and fibroblasts (Figure 4A,B). Additionally, LIV27 also showed a positive expression of human CK7 and EPCAM together with the two other CCA cell lines, HuCCT1 and WITT, in the Western blot analysis (Figure 4C). Together, these data confirm that LIV27 cells are, indeed, derived from the bile duct epithelium.

### 3.5. Karyotype Analysis

**Figure 5:**
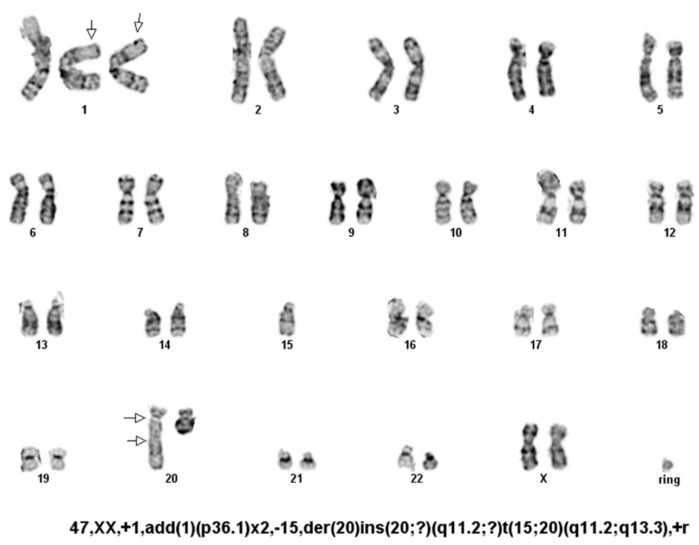
A G-banded karyotype of LIV27 cell with 47 chromosomes.

The karyotype analysis of LIV27 cells showed that the cell line is aneuploid. Each meta-phase was complex, with multiple structural and numeric abnormalities. These results are consistent with a clonal neoplastic process. A representative karyotype is shown in Figure 5, and the composite karyotype is summarized as follows: 47, XX, add(1)(p36.1) ×2,-15, der(20) ins (20;?) (q11.2;?) t (15;20) (q11.2;q13.3), +r.

### 3.6. STR Analysis

STR profiling using standard STR markers was performed for the LIV27 cell line. As expected, it revealed a female genotype. As shown in Table 1, the LIV27 cells were genotyped and found to be homozygous for the STR loci D21S11 (29), D5S818 (13), and TPOX (8), and to be heterozygous for markers TH01 (9, 9.3), D13S317 (11, 13), D7S820 (8, 14), D16S539 (8, 12), CSF1PO (10, 12), and vWA (14,19), respectively. The STR profile did not match any cell line in the ATCC, DSMZ, or JCRB cell banks, supporting the notion that it is a novel cell line.

### 3.7. Mycoplasma Detection

Mycoplasmas were not detected in the spent medium during cell culture.

## 4. Discussion

Human cancer cell lines provide excellent models for studying tumor etiology, biology, and treatment. However, only a few CCA cell lines have been established and characterized in the literature. This may be due to: (1) the low incidence of CCA; (2) the frequent late-stage clinical diagnosis of CCA, resulting in fewer surgical resections; (3) contamination with bacteria due to associated cholangitis; and (4) the difficulties in removing the abundant fibrous tissue from CCA primary cultures.

In this study, we reported on the establishment and characterization of a new CCA cell line, LIV27, derived from an intrahepatic CCA in a woman with PSC. To the best of our knowledge, this may be the first reported PSC-associated iCCA cell line. Therefore, LIV27 may provide a useful model for studying PSC-associated iCCA.

The LIV27 cells were grown continuously for over 1 month, undergoing >20 passages, and were successfully recovered after cryopreservation. The cultured cells maintained a consistent morphology from the primary culture to subsequent subculture passages. Analysis by immunofluorescence staining showed positive staining for human CK7 and CK19, consistent with the bile duct epithelial origin. Furthermore, the LIV27 cell was highly tumorigenic in nude mice, and the histology of the xenografts resembled that of the original tumor. Cytogenetic analysis confirmed that the LIV27 cells were of human origin. Cross-contamination between cell lines is a longstanding problem and a frequent cause of false experimental outcomes and scientific misinterpretation [34]. STR profiling is a simple and rapid method for cell line identification [35]. Therefore, we performed STR profiling for the LIV27 cells. A detailed STR matching analysis of this profile with the STR databases of the DSMZ, ATCC, and JCRB cell banks revealed a unique STR profile of LIV27. This method helps us to dismiss the possibility of cross-contamination between cell lines. Finally, we previously used the LIV27 cell line in several drug development studies, which confirms its usefulness for future studies [36,37].

## 5. Conclusions

In summary, we reported on the establishment and characterization of a novel human CCA cell line, termed LIV27, which was derived from a Caucasian female with PSC. This new cell line provides a new experimental model for the investigation of biological and molecular mechanisms and development of new therapeutic agents against CCA.

## Figures and Tables

**Table 1:** Short tandem repeat analysis of LIV27 cells.

STR Locus	LIV27 Cell Line
TH01	9; 9.3
D21S11	29
D5S818	13
D13S317	11; 13
D7S820	8; 14
D16S539	8; 12
CSF1PO	10; 12
AMEL	X
vWA	14; 19
TPOX	8

## Data Availability

The data presented in this study are available in the article.

## Supplementary Materials

The following supporting information can be downloaded at: https://www.mdpi.com/article/10.3390/cancers14205080/s1. All the whole western blot figures can be found in the supplementary materials.
