# Supplementary figures and images for: Establishment and Characterization of a New Human Intrahepatic Cholangiocarcinoma Cell Line LIV27
Source: Cancers (Basel). 2022 Oct 17;14(20):5080. doi: 10.3390/cancers14205080 (PMC9600735; doi:10.3390/cancers14205080)

C67  
06/24/22

250 —  
150 —  
100 —  
75 —  
50 —  
37 —  
25 —

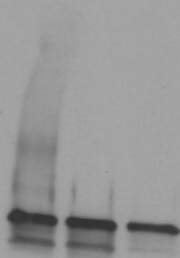

1 2 3

EPCAM

6/20/22

Super Sig

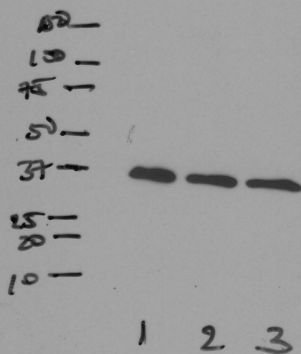

gdybdh  
6/17/22

250 —  
150 —  
100 —  
75 —  
50 —  
40 —  
25 —  
20 —  
15 —  
10 —

250 —  
100 —  
75 —  
50 —  
37 —  
25 —  
15 —  
10 —

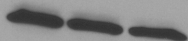

- 2' 3

Supplement: Supplementary file 1 [file cancers-14-05080-s001.zip › cancers-1950073-supplementary.pdf]
